# METTL3 inhibits primed-to-naïve transition of pluripotent stem cells through m^6^A-YTHDF2-pluripotency/*Gstp1* mRNA degradation axis

**DOI:** 10.1186/s13619-025-00241-1

**Published:** 2025-05-27

**Authors:** Sa Li, Jiajie Hao, Guangliang Hong, Hongzhi Dong, He Liu, Lingmei Jin, Zhihao Zhang, Haoyu Wu, Mingli Hu, Rujin Huang, Guanzheng Luo, Jiangping He, Jiekai Chen, Kaixin Wu

**Affiliations:** 1 Center for Biomedical Digital Science, Guangdong Provincial Key Laboratory of Stem Cell and Regenerative Medicine, Guangdong-Hong Kong Joint Laboratory for Stem Cell and Regenerative Medicine, Guangzhou Institutes of Biomedicine and Health, Chinese Academy of Sciences, Guangzhou, 510530 China; 2 School of Life Sciences, Precise Genome Engineering Center, Guangzhou University, Guangzhou, China; 3 The Fifth Affiliated Hospital of Guangzhou Medical University, Guangzhou, China; 4 Centre for Regenerative Medicine and Health, Hong Kong Institute of Science & Innovation, Chinese Academy of Sciences, Hong Kong SAR, People’s Republic of China; 5 Guangzhou Laboratory, Guangzhou, Guangdong Province 510005 China; 6 University of Chinese Academy of Sciences, Beijing, 100049 China; 7 MOE Key Laboratory of Gene Function and Regulation, Guangdong Province Key Laboratory of Pharmaceutical Functional Genes, State Key Laboratory of Biocontrol, School of Life Sciences, Sun Yat-Sen University, Guangzhou, 510275 China; 8 Guangzhou University of Chinese Medicine, Guangzhou, Guangdong 510405 China

**Keywords:** METTL3, Primed to Naïve Transition, YTHDF2, *Gstp1*

## Abstract

N6-methyladenosine (m^6^A) plays crucial roles in development and cellular reprogramming. During embryonic development, pluripotency transitions from a naïve to a primed state, and modeling the reverse primed-to-naïve transition (PNT) provides a valuable framework for investigating pluripotency regulation. Here, we show that inhibiting METTL3 significantly promotes PNT in an m^6^A-dependent manner. Mechanistically, we found that suppressing METTL3 and YTHDF2 prolongs the lifetimes of pluripotency-associated mRNAs, such as *Nanog* and *Sox2*, during PNT. In addition, *Gstp1* was identified as a downstream target of METTL3 inhibition and YTHDF2 knockout. *Gstp1* overexpression enhances PNT, whereas its inhibition impedes the transition. Overall, our findings suggest that YTHDF2 facilitates the removal of pluripotency gene transcripts and *Gstp1*, thereby promoting PNT reprogramming through m^6^A-mediated posttranscriptional control.

**Supplementary Information:**

The online version contains supplementary material available at 10.1186/s13619-025-00241-1.

## Background

N6-methyladenosine (m⁶A) modification, being the most prevalent mRNA modification, plays a pivotal role in development and cellular reprogramming (Desrosiers et al. 1974; Fu et al. 2014). Previous research has reported that METTL3, an RNA m^6^A methyltransferase, is essential for blastocyst and post-implantation developmental processes, and the absence of METTL3 can lead to defective formation of the egg cylinder (Geula et al. 2015). Studies in embryonic stem cells (ESCs) have shown that METTL3 deficiency impairs ESCs’ exit from self-renewal toward differentiation into several lineages (Batista et al. 2014; Geula et al. 2015). However, the involvement of METTL3 or m⁶A modification in cellular reprogramming is complex and context dependent. Tong Chen et al. demonstrated that microRNAs regulate m⁶A modification through sequence-pairing mechanisms, influencing METTL3 binding to target mRNAs, and increased m^6^A formation promotes cell reprogramming to pluripotency (Chen et al. 2015). Meanwhile, Wang Lab showed that ZFP217 interacts with METTL3 to restrain m^6^A modification, and low m^6^A levels in ESC-related transcripts enable pluripotency and facilitate reprogramming (Aguilo et al. 2015). In somatic cell nuclear transfer (SCNT), inefficient epigenetic remodeling hinders the recovery of totipotency, underscoring the importance of m⁶A in overcoming reprogramming barriers (Zhang et al. 2021). Collectively, these findings highlight the important role of m⁶A in cellular reprogramming.

The role of RNA m^6^A in regulating gene expression and functional execution is mostly executed by readers, the YTH domain-containing family proteins (YTHDF1-3 and YTHDC1-2) (Chen et al. 2023; Yang et al. 2023; Yang et al. 2018). In the context of somatic cell reprogramming, YTHDF2 and YTHDF3 regulate distinct RNA deadenylation pathways, thereby influencing reprogramming efficiency, and the deletion of YTHDF2 or YTHDF3 impedes reprogramming by suppressing the degradation of somatic mRNAs (Liu et al. 2020). Additionally, another m⁶A reader, YTHDC1, protects naïve pluripotency by silencing retrotransposons through m⁶A-dependent recruitment of the SETDB1-H3 K9 me3 axis, and notably, the knockout of YTHDC1 facilitates reprogramming of ESCs toward a 2-cell (2 C)-like state (Liu et al. 2021). Moreover, depletion of YTHDC1 destabilizes LINE1 RNA scaffolds, reactivating 2 C-like transcriptional programs in ESCs, a mechanism conserved in early embryos (Chen et al. 2021). It indicates that the complexity of m^6^A modification during reprogramming may be related to the roles played by different YTH families at different stages. It also underscores the need for further exploration into the role of m⁶A modification at different developmental stages.

Given METTL3’s pivotal role in post-implantation development, which aligns with the transition from the naïve to the primed pluripotent state in vivo, indicates METTL3 play a role in naïve-to-primed transition (NPT)(Geula et al. 2015). Embryonic stem cells (ESCs) and epiblast stem cells (EpiSCs) are well representing the naïve and primed states, respectively (Davidson et al., 2015; Nichols and Smith 2009; Takahashi et al. 2018; Wei et al. 2021). Investigating the reverse process, the PNT, provides valuable insights into the study of cell fate determination and the acquisition of pluripotent properties, which holds significant value for regenerative medicine (Yu et al. 2020; Yu et al. 2022). Additionally, it also holds significant implications for the manipulation of human ESCs. It is noteworthy that while both human ESCs (hESCs) and mouse ESCs (mESCs) are derived from preimplantation blastocysts, they exhibit substantial differences in several key characteristics (Davidson et al. 2015). Interestingly, hESCs share more similarities with mouse EpiSCs than with mESCs, suggesting that the properties of mouse EpiSCs are more comparable to those of human pluripotent cells (Messmer et al. 2019; Schnerch et al. 2010). The isolation and study of mouse EpiSCs, as well as the examination of the naïve and primed pluripotency states in these cells, provide crucial theoretical insights into the unique features of human pluripotent stem cells.

In this study, we utilized STM2457, an enzymatic inhibitor of RNA m^6^A methyltransferase METTL3, and found METTL3 inhibition significantly promote the efficiency of PNT. Further, we discovered that knockout of YTHDF2, but not YTHDF1 or YTHDF3, in EpiSCs led to a significant m^6^A-dependent increase in PNT efficiency. Mechanistically, we found that *Ythdf2* deficiency not only suppressed the mRNA clearance of pluripotency-related genes (such as *Nanog* and *Sox2*), but also resulted in elevated expression of *Gstp1*—a glutathione S-transferase known for its role in detoxification and antioxidant defense—which promotes PNT independently of its canonical detoxification functions (Semprasert et al. 2024; Wang et al. 2023).

## Results

### METTL3 intervention promotes PNT in an m^6^A-dependent manner

**Fig. 1:**
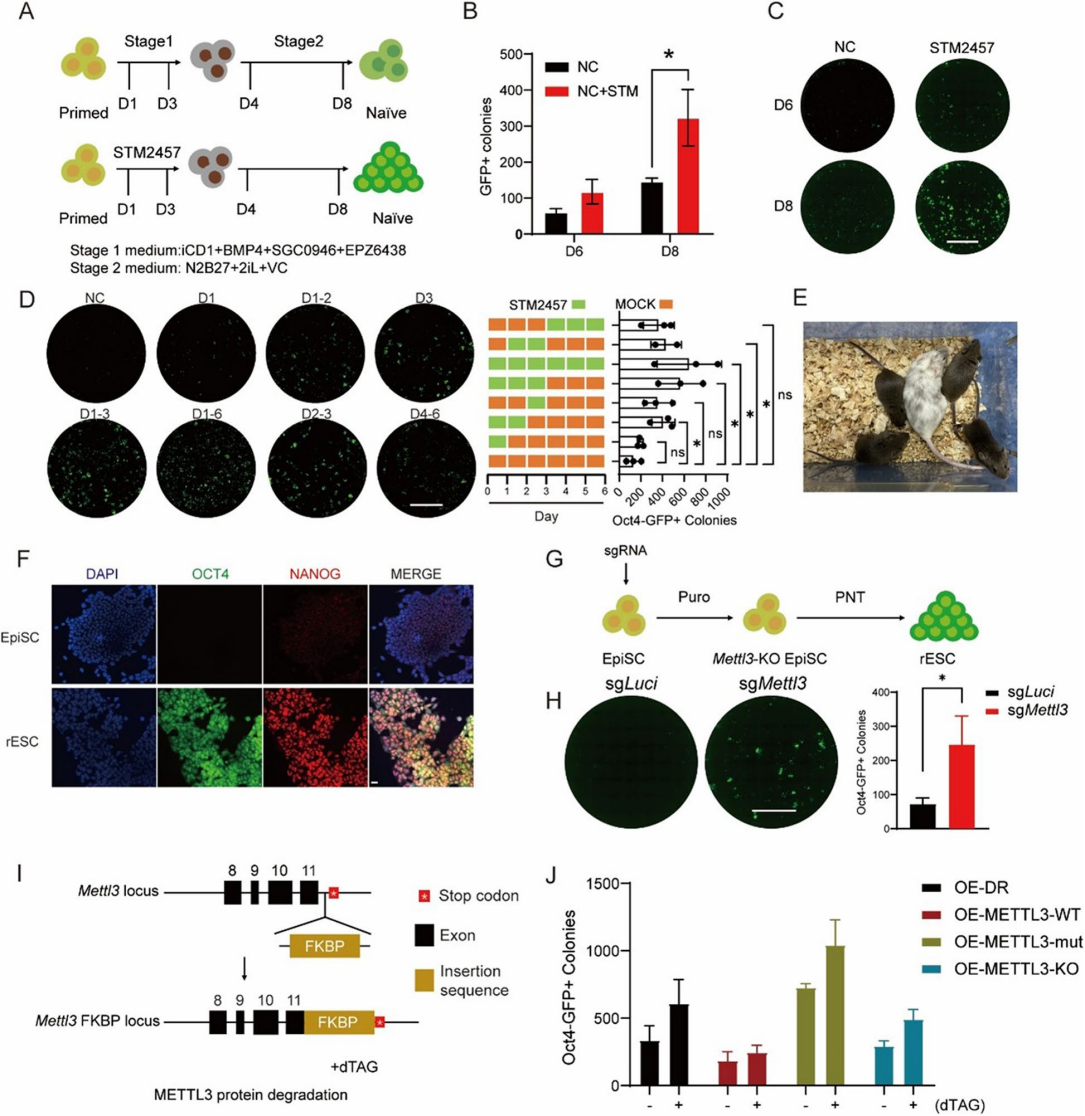
METTL3 inhibition advances PNT depending on m.^6^A. **A** Schematic illustration of the BiPNT system and STM2457 treatment strategy. The reprogramming medium iCD1 contains DMEM supplemented with N-2, B-27, vitamin C (Vc), LiCl, bFGF, CHIR99021 (CHIR) and LIF. **B** Quantification of GFP + colonies at day 6 and day 8 of PNT with or without STM2457 treatment. (*n* = 3, error bar, s.d; unpaired t-test, *p < 0.05, **p < 0.01, ***p < 0.001). **C** Oct4-GFP fluorescent scanning image of whole 24-well plates showing GFP + colonies at day 6 and day 8 of PNT with or without STM2457 treatment. Scale bars, 5 mm. **D** Left: Oct4-GFP fluorescent scanning image of whole 24-well plates showing PNT efficiency with STM2457 treatment at different stages. Scale bars, 5 mm. Right: Quantification of GFP + colonies. Data are mean ± s.d., *n* = 3 independent experiments. **E** Representative images of chimaeric mice generated from STM2457-treated rESCs showing successful germline transmission. **F** Immunofluorescence analysis of naïve pluripotency markers OCT4(ΔPE-promoter) and NANOG in EpiSCs and STM2457-treated rESCs. Scale bars, 20 μm. **G** Schematic representation of CRISPR-Cas9-mediated *Mettl3* knockout strategy in the PNT system. **H** Left: Oct4-GFP fluorescent scanning image of whole 24-well plates showing PNT efficiency in cells with CRISPR-Cas9-mediated knockout of *Mettl3* or *luciferase* (control). Scale bars, 5 mm. Right: Quantification of GFP + colonies. Data are mean ± s.d., *n* = 3 independent experiments. **I** Schematic representation of METTL3-FKBP-dTag strategy in the PNT system. Black boxes represent exons, with exon 11 being the final exon of *Mettl3*. The red star denotes the stop codon. The FKBP sequence was inserted between the last exon and the stop codon of *Mettl3*. **J** PNT efficiency in METTL3-deficient and rescue experiments. (*n* = 6, error bar, s.d)

RNA N6-methyladenosine (m^6^A) modification plays a crucial role in cell fate determination (Liu et al. 2021; Liu et al. 2020). However, its precise function in regulating the PNT remains elusive. To explore the role of m^6^A in PNT, we utilized STM2457, an enzymatic inhibitor of RNA m^6^A methyltransferase METTL3 within the BMP4- induced PNT (BiPNT) system (Yankova et al. 2021). This system utilizes OG2 cells harboring a homozygous *Oct4* (ΔPE-promoter)-GFP construct, where “ΔPE-promoter” denotes an *Oct4* promoter lacking the proximal enhancer (PE). As a result, the expression of the *Oct4*-GFP reporter is independent of the proximal enhancer and is solely driven by activation of the distal enhancer (DE) of *Oct4*, leading to GFP expression. We found that STM2457 treatment significantly enhanced PNT efficiency in the BiPNT system (Fig. 1A-C, Fig. S1A, B)(Yu et al. 2020). Additionally, m^6^A ELISA assays demonstrated that STM2457 treatment significantly decreased m^6^A mRNA levels (Fig. S1C). To elucidate the specific stage of PNT where METTL3 exerts its effects, we introduced STM2457 treatment at various stages of PNT and observed a promotional effect across all periods (Fig. 1D). However, the impact was most pronounced during the early phase (e.g., days 1~3), suggesting that METTL3 predominantly influences the initial stages of PNT. Meanwhile, we have determined that the optimal concentration of STM2457 for our study was 10 nM (Fig. S1D, E). We next tested the impact of METTL3 inhibition on various PNT condition within the BiPNT system to investigate its robust effect across different experimental conditions. Notably, STM2457 exhibited a consistent promotional effect across these systems, particularly under -EZP conditions (Fig. S1F, G), suggesting that its influence on PNT is independent of system-specific small molecules. It is worth noting that the cells derived from METTL3 inhibition during PNT exhibited high quality and could establish cell lines. Immunofluorescence assay revealed a high expression of the naïve pluripotency genes in the cells that had completed PNT, which are also referred to as reset embryonic stem cells (rESCs), and these rESCs giving rise to chimaera are capable of germline transmission (Fig. 1E, F).

To mitigate potential off-target effects of pharmacological inhibition, we employed two complementary genetic approaches. First, using a CRISPR-Cas9-mediated *Mettl3* gene knockout system (Fig. 1G, Fig. S1H), we observed enhanced PNT efficiency, corroborating the effects observed with STM2457 treatment. (Fig. 1H). We further validated these findings using a METTL3-FKBP/dTAG protein degradation system (Fig. 1I, Fig. S1I), and to determine whether the functions of METTL3 during reprogramming occur in an m^6^A-dependent manner, we overexpressed wild-type (WT) or catalytically dead mutants (DPPW 395–398 APPA) of METTL3. Our findings indicate that the promotive effect on PNT depends on METTL3’s catalytic activity, highlighting the crucial role of METTL3’s m^6^A methyltransferase function during PNT (Fig. 1J).

In summary, our findings demonstrate that METTL3-mediated m^6^A modification acts as a critical regulator of the primed-to-naïve transition, with its inhibition consistently enhancing PNT efficiency in high quality across various experimental systems and conditions.

### METTL3 inhibition accelerates PNT with rapid activation of pluripotent genes

**Fig. 2:**
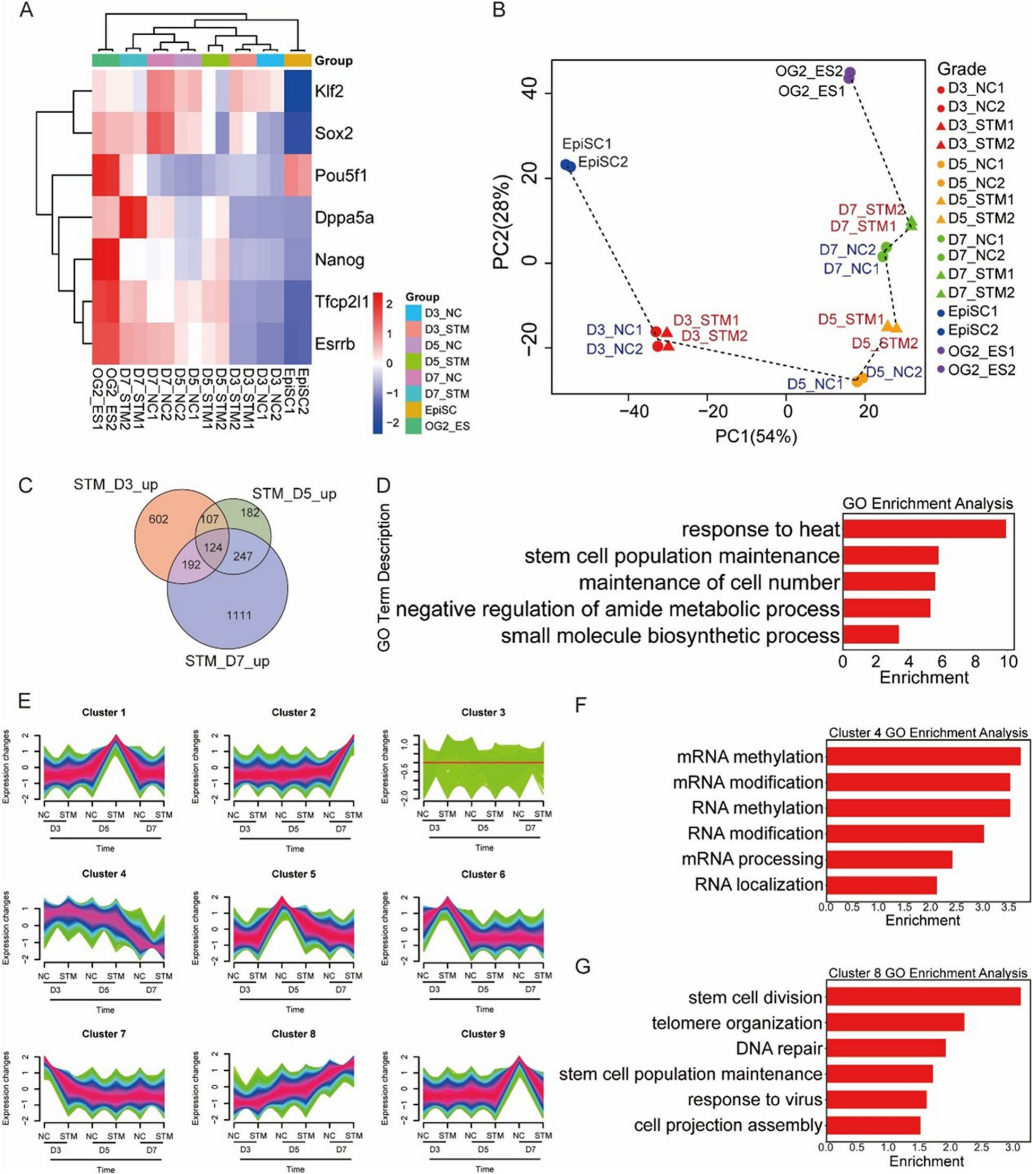
METTL3 inhibition expedites PNT through the rapid activation of pluripotent genes. **A** Heatmap showing expression dynamics of key pluripotency genes during PNT (days 3, 5, and 7) with or without STM2457 treatment. **B** Principal component analysis (PCA) of RNA-seq data from PNT samples with or without STM2457 treatment. Dotted line indicate the trajectory of transcriptional changes during PNT. *n* = 2 biological replicates per condition. **C** Venn diagram showing overlap of upregulated genes at days 3, 5, and 7 of PNT following STM2457 treatment (fold change > 1.5, *P* < 0.05). **D** Gene Ontology (GO) analysis of commonly upregulated genes identified in c. **E** Mfuzz clustering analysis revealing nine distinct gene expression patterns during PNT. Shown are mean expression trajectories for each cluster. **F** GO analysis of genes in cluster 4 showing enrichment of RNA modification pathways. **G** GO analysis of genes in cluster 8 showing enrichment of pluripotency maintenance pathways

To gain insights into the specific mechanisms of METTL3 inhibition during PNT, we conducted RNA sequencing (RNA-seq) on PNT with STM2457 treatment at days 3/5/7. Analysis of the RNA-seq data showed that STM2457 treatment consistently led to higher expression of naïve pluripotency genes, with virtual trajectory mapping indicating an accelerated PNT process (Fig. 2A, B, Fig. S2A, B), which was also confirmed by the results of RT-qPCR (Fig. S2C). Furthermore, to find the downstream effect regulator of METTL3 inhibition, we intersected the genes upregulated at D3/5/7 in PNT and conducted Gene Ontology (GO) analysis. Notably, pluripotency maintenance pathway and cell division were enriched, which was positive to naïve reprogramming (Fig. 2C, D).

Additionally, we performed mfuzz analysis on the sequencing data following the virtual trajectory order of D3/5/7 with and without STM2457 treatment, as shown in Fig. 2B. This analysis revealed nine distinct clusters (Fig. 2E, Fig. S2D), among which cluster 4 exhibited consistent downregulation throughout the process. GO analysis of cluster 4 demonstrated enrichment in RNA modification-related pathways (Fig. 2F). Conversely, cluster 8 showed consistent upregulation and was enriched in pluripotency maintenance pathways, which is consistent with the previous results of METTL3 inhibition promoting pluripotency expression (Fig. 2G).

In summary, our transcriptome analysis further substantiates our experimental findings that METTL3 inhibition promotes PNT through coordinated suppression of RNA modification pathways and enhancement of pluripotency maintenance programs, reinforcing the crucial regulatory role of m^6^A modification in primed-to-naïve transition.

### Loss of the m⁶A reader protein YTHDF2 promotes PNT

**Fig. 3:**
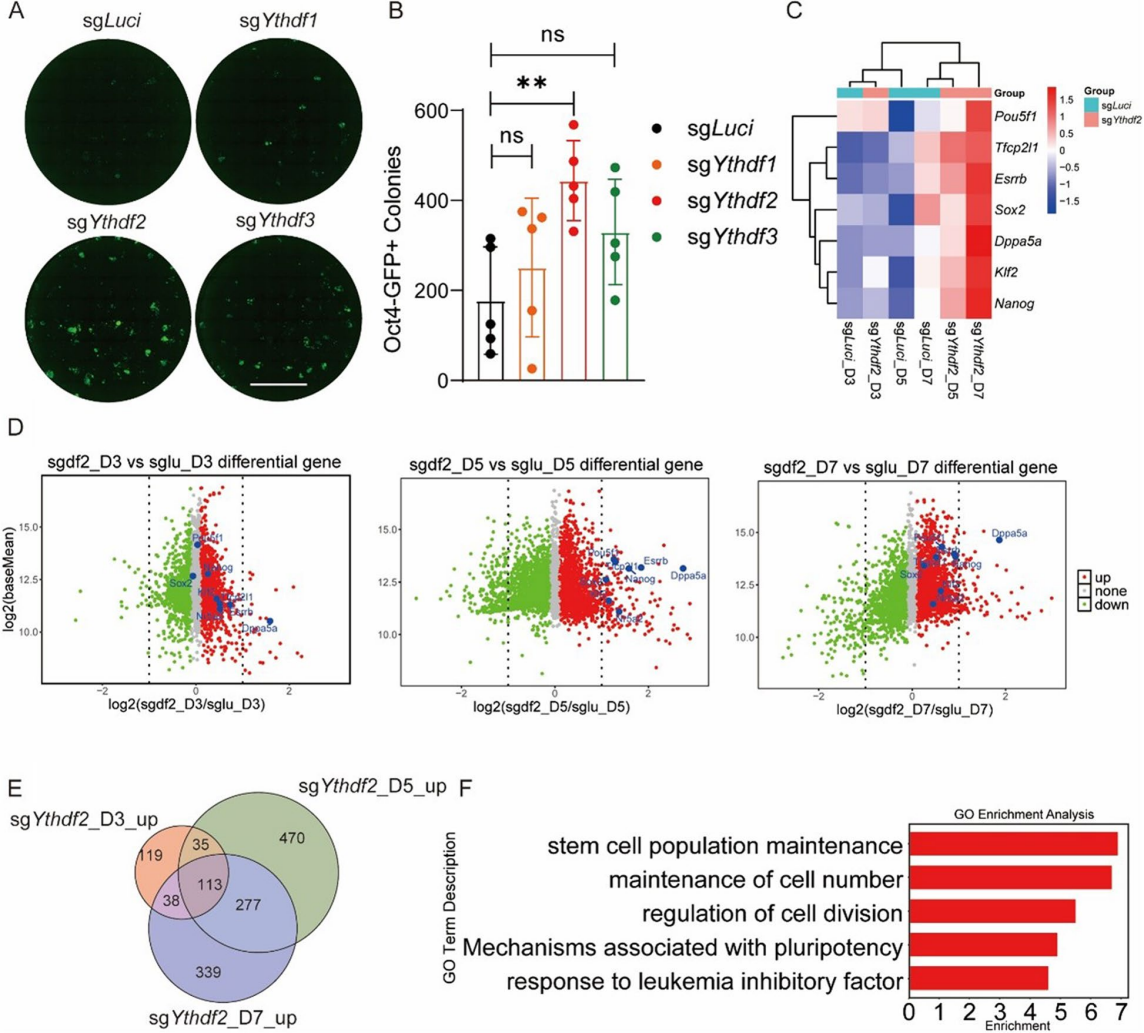
Loss of the m⁶A reader protein YTHDF2 promotes PNT. **A** Oct4-GFP fluorescent scanning image of whole 24-well plates showing PNT efficiency in CRISPR-Cas9-mediated *Ythdf1, Ythdf2, and Ythdf3* knockout cells at day 6. Scale bars, 5 mm. **B** Quantification of GFP + colonies in YTHDF-deficient cells during PNT. (*n* = 5, error bar, s.d; unpaired t-test, *p < 0.05, **p < 0.01, ***p < 0.001). **C** Heatmap showing expression dynamics of naïve pluripotency genes during PNT (days 3, 5, and 7) in control and *Ythdf2*-knockout cells. **D** Time-course RNA-seq analysis showing differential expression of key pluripotency factors in control versus *Ythdf2*-knockout cells during PNT. **E** Venn diagram depicting overlap of upregulated genes at days 3, 5, and 7 of PNT in *Ythdf2*-knockout cells compared to control. **F** GO analysis of commonly upregulated genes identified in E

To identify the specific m^6^A reader responsible for the executory role of RNA m^6^A during PNT, we carried out a comprehensive series of CRISPR-Cas9-mediated knockout experiments targeting *Ythdf1*, *Ythdf2*, and *Ythdf3*. Notably, YTHDF2 deficiency emerged as the most potent effector of PNT (Fig. 3A, B, Fig. S3A). Subsequently, through a detailed analysis of gene expression profiles in YTHDF2-deficient cells during PNT at multiple time points (D3/5/7), we uncovered a consistent pattern of increased expression of naive pluripotency genes throughout the transition process after *Ythdf2* knockout (Fig. 3C, D), RT-qPCR also confirmed the upregulation of naïve pluripotency genes at D7 following *Ythdf2* knockout (Fig. S3B). To elucidate the stage-specific regulatory mechanisms of YTHDF2 during PNT, we overlapped the up-regulated genes following YTHDF2 deficiency at days 3, 5, and 7, and further performed gene ontology analysis. Specifically, pathways related to cell division and pluripotency maintenance related pathways were enriched, which is consistent with the results of METTL3 inhibition (Fig. 3E, F). Furthermore, we utilized the mfuzz algorithm to identify clusters with similar expression patterns during PNT (Fig. S3C). We then selected cluster 9, characterized by a continuous up-regulation of gene expression, for deeper analysis. Consistently, genes in cluster 9 were also found to be involved in pathways related to pluripotency maintenance (Fig. S3D). Collectively, these findings reveal a critical role of the YTHDF2 in regulating pluripotent genes in primed-to-naïve transition.

### Naïve pluripotent gene mRNAs are major target of YTHDF2-mediated degradation in PNT

**Fig. 4:**
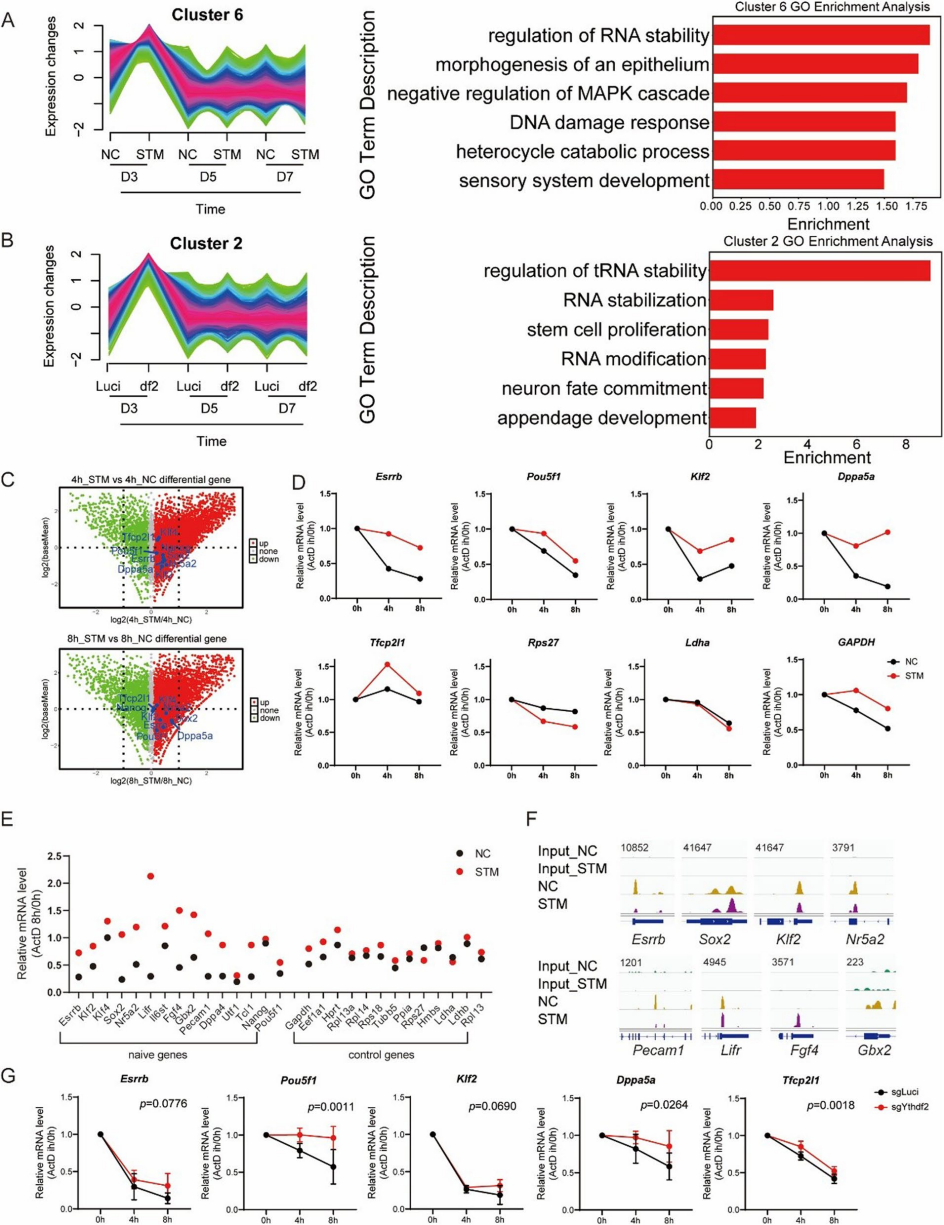
mRNAs of naïve pluripotent genes are major targets of YTHDF2-mediated degradation within PNT. **A** GO Analysis of cluster 6 genes in Fig. 2E. **B** GO Analysis of cluster 2 genes in Fig. S3C. **C** mRNA lifetime analysis of RNA-seq data in D3 PNT with or without STM Treatment, Cells were treated with actinomycin D (ActD) and samples were collected at 0 h, 4 h, and 8 h. Red dots represent genes with inhibited degradation. Naïve pluripotent genes are labeled. **D** mRNA lifetime analysis of selected genes from RNA-seq data in D3 PNT with or without STM Treatment, Cells were treated with Actinomycin D (ActD) and samples were collected at 0 h, 4 h, and 8 h. **E** mRNA lifetime analysis of naive pluripotency genes and housekeeping genes from RNA-seq data, measured in D3 PNT with or without STM Treatment, each for control (black dots) and STM Treatment (red dots). **F** m^6^A peak coverage on specific naive pluripotent transcripts and housekeeping transcripts in PNT m6 A-RIPseq data. **G** mRNA lifetime of selected naive pluripotency genes in *Ythdf2* knockout and control cells, measured by RT-qPCR. Cells were treated with actinomycin D (ActD) and samples were collected at 0 h, 4 h, and 8 h. (*n* = 4, error bar, s.d; Two-way ANOVA with p-values calculated for the column factor)

YTHDF2 is a well-known factor involved in mRNA degradation. It binds to m^6^A modified mRNAs and recruits the CCR4-NOT deadenylase complex, leading to the shortening of the poly(A) tail and subsequent mRNA degradation (Du et al. 2016; Liu et al. 2020). This process is crucial for regulating gene expression and various cellular functions. Given these considerations, we hypothesize that m^6^A-marked naive pluripotent transcripts undergo accelerated degradation mediated by YTHDF2. Correspondingly, we found up-regulated genes during the early stages of PNT following METTL3 inhibition or *Ythdf2* knockout are enriched in pathways related to RNA stability (Fig. 4A, B).

To validate this hypothesis experimentally, we investigated the impact of STM2457 treatment on mRNA stability utilizing actinomycin D (ActD), a transcription inhibitor. We found that STM2457 treatment markedly enhanced the stability of naïve gene mRNAs, demonstrating increased resistance to degradation (i.e., *Esrrb*, *Pou5f1* and *Nanog*) (Fig. 4C, D, Fig. S4A). Additionally, analysis of published RNA m^6^A RIP datasets from ESCs revealed prevalent m^6^A modifications on naive gene transcripts but not on housekeeping genes. (Fig. S4B). Further examination revealed that STM2457 treatment significantly inhibited degradation of naive gene transcripts while having minimal effect on housekeeping transcripts, correlating with their m^6^A modification patterns (Fig. 4E, Fig. S4B). Meanwhile, we performed m^6^A-RIPseq analysis on PNT samples treated with either NC or STM2457 at day 3. The analysis revealed that m^6^A levels on most key naive transcripts were downregulated following STM2457 treatment, consistent with our previous findings (Fig. 4F).

To further substantiate the role of m^6^A mRNA degradation in PNT, we examined mRNA stability in *Ythdf2* knockout conditions during PNT. Notably, *Ythdf2* knockout was shown to enhance the stability of naive pluripotent transcripts (Fig. 4G), which is consistent with the results of METTL3 inhibition. We also assessed the expression level of primed markers during PNT. Although STM2457 treatment and *Ythdf2* knockout led to a slight upregulation, the initial expression levels were quite low and insufficient to significantly affect PNT (Fig. S4C, D). Additionally, we investigated the effect of METTL3 inhibition on the ESC-to-EpiSC transition (NPT) and found an inhibitory effect (Fig. S4E). This may be due to its role in downregulating naive genes (Fig. S4F), which was also supporting its function in maintaining the naïve pluripotent state.

In summary, these analyses demonstrate that both METTL3 inhibition and *Ythdf2* deficiency enhance PNT through the stabilization of naïve pluripotent gene mRNAs, primarily via modulation of m^6^A mRNA degradation pathways by YTHDF2.

### *Gstp1* is a key mediator in the Primed-to-Naïve transition regulatory network

**Fig. 5:**
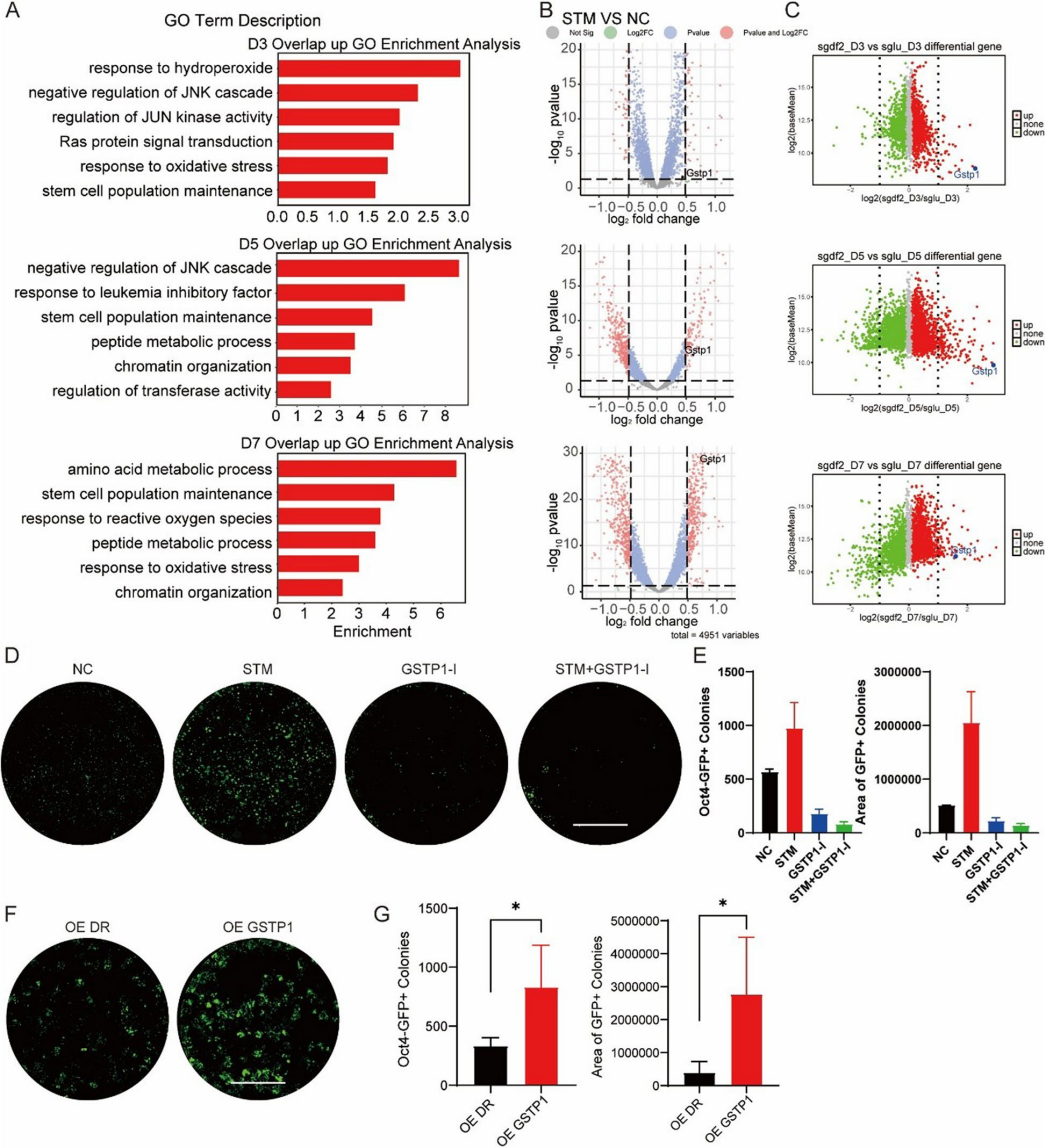
*Gstp1* functions as a crucial mediator within the PNT regulatory network. **A** GO Analysis of Co-upregulated Genes in Two Batches of Sequencing Data: PNT RNA-seq with or without STM and PNT RNA-seq with *Ythdf2* or Luciferase Knock-out. **B** Dot plot showing differentially expressed genes (DEGs) from RNA-seq results of D3/5/7 PNT, with or without STM2457 treatment. *Gstp1* gene are labeled. **C** Dot plot showing differentially expressed genes (DEGs) from RNA-seq results of D3/5/7 PNT, PNT cells were genetically modified via CRISPR/Cas9 to knock out *Ythdf2* or *luciferase* (as a Control) Genes. Red dots represent upregulated genes and green dots represent downregulated genes. *Gstp1* gene are labeled. **D** Fluorescence Images of PNT Treated with STM, GSTP1-I, STM + GSTP1-I, and Control Respectively. Scale bars, 5 mm. **E** Number and area of GFP + colonies of D7 PNT Treated with STM, GSTP1-I, STM + GSTP1-I, and Control Respectively. (*n* = 2, error bar, s.d; unpaired t-test, *p < 0.05, **p < 0.01, ***p < 0.001). **F** Oct4-GFP fluorescent scanning image of whole 24-well plates at day 7 of PNT with overexpressed GSTP1 or DsRed(DR) as a control. Scale bars, 5 mm. **G** Number and area of GFP + colonies of D7 PNT with overexpressed GSTP1 or DS-RED. (*n* = 4, error bar, s.d; unpaired t-test, *p < 0.05, **p < 0.01, ***p < 0.001)

Beyond the role of RNA m^6^A in regulating pluripotency-associated mRNAs during PNT, we were curious about other potential RNA m^6^A regulatory targets. To explore this possibility, we conducted an overlap analysis of RNA-seq datasets from both STM2457 treatment and *Ythdf2* deficiency conditions. Through systematic analysis of the sequencing data across D3/5/7 timepoints, multiple signaling pathways showed significant enrichment. Intriguingly, JNK-related pathways emerged as a common theme in both D3 and D5 datasets (Fig. 5A), suggesting a potential role for JNK signaling in the PNT process. When examining the specific gene components within these enriched pathways, we discovered that *Gstp1*, a key negative regulator of JNK1/2, appeared with the highest frequency among the identified genes. This observation prompted us to examine *Gstp1*’s differential expression patterns in the PNT sequencing data under both STM2457 treatment and *Ythdf2* knockout conditions. Remarkably, *Gstp1* exhibited significant upregulation across all three timepoints (D3/5/7) in both experimental conditions (Fig. 5B, C), indicating a potentially crucial role for *Gstp1* in the PNT process.

To further elucidate the regulatory mechanisms governing *Gstp1* expression, we examined the published RNA m^6^A RIP datasets from ESCs and discovered the presence of m^6^A modifications on the transcripts of *Gstp1* gene (Fig. S5A). Then, we analyzed ActD sequencing data from STM2457-treated samples. This analysis revealed a slight inhibition of *Gstp1* degradation in the STM2457 treatment group (Fig. S5B). Correspondingly, we observed a similar phenomenon of inhibited *Gstp1* degradation under *Ythdf2* knockout conditions (Fig. S5C). These findings suggest that *Gstp1* expression is slightly affected by the suppression of YTHDF2-mediated mRNA degradation, indicating that YTHDF2 fine-tunes *Gstp1* levels during the PNT.

Subsequently, we investigated the specific role of GSTP1 in PNT using Ezatiostat, a selective inhibitor of GSTP1-1 catalytic activity. Our experiments demonstrated that administering the GSTP1 inhibitor during the first three days significantly suppressed PNT efficiency, with both 1 μM and 10 μM concentrations proving effective (Fig. 5D, E; Fig. S5D, E). Notably, this inhibitory effect also extended to STM2457-treated PNT samples (Fig. 5D, E), suggesting that *Gstp1* may function downstream of STM2457 in the regulatory pathway. Additionally, following STM2457 treatment, we knocked down *Gstp1*, reducing its expression to levels comparable to the control (Fig. S5F). Consistent with this, *Gstp1* knockdown also resulted in a decrease in PNT efficiency, thereby reversing the enhancement observed with STM2457 treatment (Fig. S5G). To further validate this finding, we overexpressed *Gstp1* during the process of PNT (Fig. S5H). Consistent with our hypotheses, this intervention led to an enhancement in PNT efficiency (Fig. 5F, G), thereby corroborating the pivotal role of *Gstp1* in facilitating the PNT.

All in all, these findings demonstrate that *Gstp1* serves as a key mediator in PNT regulation, with its expression modulated through the METTL3-m^6^A-YTHDF2 axis.

## Discussion

**Fig. 6:**
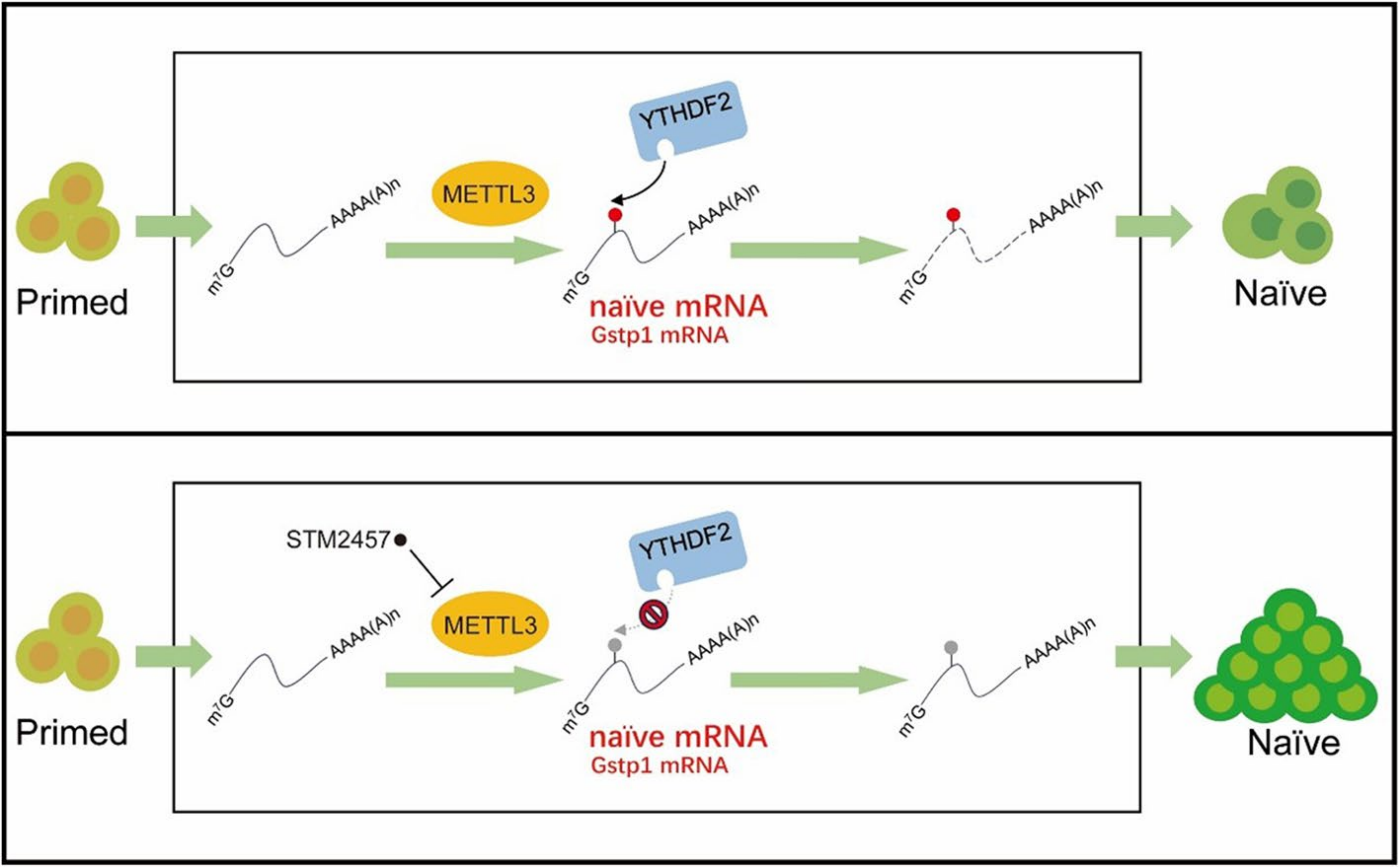
Schematic diagram of the regulatory mechanism of METTL3-m^6^A-YTHDF2 axis in PNT. Upper panel: In conventional PNT, METTL3 catalyzes the establishment of m^6^A modifications on naive mRNA and *Gstp1* mRNA, as indicated by red dots. These m^6^A-modified transcripts are subsequently recognized by YTHDF2, which mediates their degradation. Lower panel: Under STM2457 treatment during PNT, METTL3 inhibition prevents the establishment of m^6^A modifications on naive mRNA and *Gstp1* mRNA (gray dots indicate the absence of m^6^A modifications), ultimately impeding the degradation of these transcripts. Similarly, disruption of YTHDF2-mediated recognition of m^6^A (e.g., via *Ythdf2* knockout) produces the same effect. In addition, the short green arrow represents the PNT workflow, while the long green arrow represents the RNA dynamics during the PNT process

In this study, we demonstrate that METTL3-mediated m^6^A modification functions as a critical regulatory mechanism in the transition from primed to naïve pluripotency. RNA m^6^A modification plays crucial roles in various aspects of stem cell fate determination, including somatic cell reprogramming and embryonic development (Geula et al. 2015; Liu et al. 2020). While the detailed regulatory mechanisms remain largely unexplored, we utilized the well-established developmental cell fate transition model PNT to reveal that inhibition of METTL3, either through pharmacological intervention or genetic manipulation, significantly promotes PNT efficiency. Our study provides mechanistic insight into how the m^6^A pathway specifically regulates PNT (Fig. 6).

Specifically, our results identify YTHDF2, but not YTHDF1 and YTHDF3, as the primary m^6^A reader protein mediating the effects of m^6^A modification during PNT. Unlike METTL3 inhibition, which prevents the establishment of m^6^A modifications on specific mRNAs, the loss of YTHDF2 impairs the recognition and binding of m^6^A on these transcripts. Despite their mechanistic differences, both scenarios lead to the same outcome, disrupting the downstream functional effects of m^6^A modifications. Specifically, the specific role for YTHDF2 in PNT suggests a unique regulatory mechanism involving targeted mRNA degradation. Furthermore, we observed that YTHDF2 mediates the degradation of naive gene mRNAs during PNT, thus serving as a critical regulatory factor in this process. A key mechanistic finding of our study is the identification of *Gstp1* as a crucial downstream effector in the METTL3-m^6^A-YTHDF2 regulatory axis. While GSTP1 is traditionally associated with detoxification and antioxidant functions, intriguingly, it can also inhibit JNK activity through complex formation (Shen et al. 2025). Previous studies have shown that JNK downstream target c-jun impedes PNT (Li et al. 2023), suggesting that m^6^A-mediated regulation of *Gstp1* may serve as a molecular switch controlling JNK activity during PNT, thereby influencing the expression of downstream targets of the Jun transcription factor family. This regulatory circuit represents a previously unrecognized mechanism by which post-transcriptional modification can influence cell signaling pathways during cell fate transitions.

Moreover, several limitations of our study should be noted. Despite repeated CRISPR–Cas9 experiments, we regrettably failed to achieve complete knockout of *Mettl3*. Although we have identified GSTP1 as a key effector, both METTL3 inhibition and *Ythdf2* knockout only slightly suppress *Gstp1* transcript degradation. Furthermore, our PNT m^6^A RIP-seq data did not show any m^6^A enrichment on *Gstp1* transcripts, even though MeRIP data from other laboratories showed m^6^A enrichment on *Gstp1* transcripts, this discrepancy may be due to differences in experimental stability. While it remains uncertain whether *Gstp1* mRNA is regulated by m^6^A, our results confirm that GSTP1 is indeed a factor that promotes PNT. In addition, there may be additional downstream targets of YTHDF2 that contribute to PNT regulation. The downstream mechanisms by which GSTP1 promotes PNT also require further investigation. Moreover, the precise temporal dynamics of m^6^A modification during PNT remain to be fully elucidated. Future studies utilizing high-resolution temporal profiling of m^6^A modifications could provide additional insights into the regulatory mechanisms controlling cell fate transitions. Additionally, while our study demonstrates the importance of METTL3 mediated m^6^A modification in PNT, the potential contributions of other m^6^A writers and erasers remain to be explored. The interplay between different components of the m^6^A regulatory machinery during cell fate transitions represents an important area for future investigation.

In conclusion, our study establishes the m^6^A-YTHDF2-pluripotency mRNA degradation axis as a critical regulator of PNT and identifies GSTP1 as a key downstream effector. These findings not only advance our understanding of the molecular mechanisms controlling pluripotent state transitions but also suggest potential therapeutic strategies for manipulating cell fate in regenerative medicine applications.

## Materials and methods

### Mice

129 Sv/Jae and ICR mice were purchased from Beijing Vital River Laboratory, all animal experiments were carried out in compliance with the Animal Protection Guidelines set forth by the Guangzhou Institutes of Biomedicine and Health, Chinese Academy of Sciences, Guangzhou, China.

### Cell lines and cell culture condition

Mouse Oct4-GFP EpiSCs were generously provided by Dr. Shengyong Yu’s laboratory. Stable EpiSCs were cultured in feeder-free conditions on dishes coated with fetal bovine serum (FBS). The culture medium, referred to as FAX, consisted of N2B27 supplemented with bFGF (15 ng/ml, PeproTech), Activin A (20 ng/ml, PeproTech), and XAV939 (1 μM, Selleck).The N2B27 medium was prepared by combining equal parts of DMEM/F12 and Neurobasal (both from GIBCO), with the following additives: 0.5% N2, 1% B27, 1% GlutaMAX, 1% non-essential amino acids (NEAA), and 0.1 mM β-mercaptoethanol (all from GIBCO).For passaging, EpiSCs were dissociated using Accutase (Sigma) and seeded as single cells in FAX medium supplemented with 5 μM Y27632 (Selleck). Approximately 10,000 cells were plated in each well of a 24-well plate, with passaging occurring every 3 days. To maintain optimal growth conditions, the culture medium was replaced daily.

Feeder-free culture conditions were employed for maintaining both mouse ESCs and rESCs. These cells were grown on surfaces coated with 0.2% gelatin, utilizing N2B27 medium enriched with 2i/LIF. The 2i/LIF supplement consisted of PD0325901 (1 µM, synthesized at GIBH), CHIR99021 (3 µM, synthesized at GIBH), and LIF (1,000 U ml − 1, Millipore). For routine subculture, a solution of 0.05% trypsin–EDTA (GIBCO) was used to dissociate the cells. This passaging procedure was performed at 3-day intervals, ensuring optimal cell density and growth conditions. The culture system described above provided an environment conducive to maintaining the pluripotent state of both mouse ESCs and rESCs, while the regular passaging schedule facilitated consistent cell proliferation and experimental reproducibility.

### Cell culture medium

Stage 1 Medium:

- Base: iCDl medium (DMEM, 0.5 × N2, 0.5 × B27, 1% GlutaMAX, 1% NEAA, 1% sodium pyruvate, 0.1 mM 2-mercaptoethanol). Supplements: Vitamin C (50 μg/ml), bFGF (10 ng/ml), LiCl (5 mM), LIF (1,000 U/ml), CHIR99021 (1 μM), BMP4 (10 ng/ml), SGC0946 (1 μM), EPZ6438 (2.5 μM).

Stage 2 Medium (N2B27-2iL):

- Base: N2B27 medium. Supplements: Vitamin C (50 μg/ml), LIF (1,000 U/ml), PD0325901 (1 μM), CHIR99021 (3 μM).

Cell Culture Protocol:

- Dissociate EpiSCs into single cells using Accutase, seed 5,000 cells per well in a 24-well plate pre-coated with FBS. Use N2B27 + FAX medium supplemented with 5 μM Y27632. On day 1, replace the initial medium with Stage 1 medium.

Refresh Stage 1 medium daily for 3 days. After 3 days, switch to Stage 2 medium (N2B27-2iL). Refresh Stage 2 medium daily for the remaining 5 days of culture.

### CRISPR-Cas9-mediated gene knockout

*Ythdf1*, *Ythdf2*, *Ythdf3*, and *Mettl3* knockout (KO) EpiSC cells were generated using the lentivirus-mediated CRISPR-Cas9 technology. The sgRNA oligos for target genes were annealed and cloned into the BsmB1 (Thermo Fisher Scientific) digested plasmid lentiCRISPR v2 vector (52961, Addgene, Cambridge, MA). To knockout target genes, EpiSC cells were transiently transfected with lentiCRISPR-v2 vector carrying respective sgRNAs and selected with puromycin (puro; 1 ug/ml for EpiSC cells) at low density in 24-well plates for 5~7 days. Colonies were amplified and validated for KO by immunoblots. sgRNA sequences are provided in Supplementary table “sgRNA” section.

### siRNA transfection

For *Gstp1* knockdown experiments, synthetic siRNA targeting *Gstp1* (siGstp1) and a non-targeting negative control siRNA (siNC) were purchased from IGE Biotechnology (Guangzhou, China). Cells were transfected with siRNA using Lipofectamine 3000 (Invitrogen) according to the manufacturer’s instructions. Transfection was performed at a final siRNA concentration of 50 nM, and cells were harvested 72 h post-transfection for subsequent analyses. siRNA sequences are provided in Supplementary table “siRNA” section.

### Western blot

Following standard laboratory protocols, Western blotting analysis was performed. PVDF membranes underwent blocking in 5% non-fat milk solution prior to overnight incubation with primary antibodies at 4 °C. We employed the following primary antibodies: YTHDF1 (17479–1-AP, Proteintech), YTHDF2 (ARP67917_P050, Vivasysbio), YTHDF3 (25537–1-AP, Proteintech), and METTL3 (15073–1-AP, Proteintech). Subsequently, membranes were subjected to washing with TBS containing 1% Tween-20, followed by room temperature incubation with HRP-conjugated secondary antibodies (goat anti-rabbit or goat anti-mouse) for 1 h. Signal visualization was accomplished using an ECL kit (P10300, NCM), while band images were documented using a Bio-Rad imaging system. The complete antibody list is provided in Supplementary table “Antibody” section.

### Flow cytometry

To prepare flow cytometry single-cell suspensions, cells underwent treatment with 0.25% trypsin–EDTA and were harvested through centrifugation. Following PBS washing, we resuspended the cell pellet in PBS supplemented with 0.1% BSA. The suspension was passed through a 70 μm membrane filter. Cell analysis was conducted using an Accuri C6 flow cytometer (BD Biosciences), with GFP fluorescence measured via the FITC channel. Data processing was completed using FlowJo version 7.6.1.

### Total RNA extraction and qRT-PCR

Using TRIZOL reagent (MRC), total RNA was isolated from cell lines. We determined RNA concentration and purity using a NanoDrop One C spectrophotometer (ND-ONEC-W, Thermo Fisher Scientific), selecting only samples with 260/280 ratios ranging from 1.8 to 2.0. For cDNA synthesis, 1 μg of RNA was reverse transcribed using HiScript II Q RT SuperMix for qPCR (R222-01, Vazyme). Subsequently, the diluted cDNA served as template for qPCR reactions utilizing ChamQ SYBR qPCR Master Mix (Q311-02, Vazyme). All gene-specific primers have been documented in the online appendix. The gene-specific primer sequences are provided in Supplementary Table “qPCR Primer” section.

### Immunofluorescence

Cells grown on coverslips were washed with PBS and subjected to fixation using 4% paraformaldehyde for 30 min at room temperature. The permeabilization step involved a blocking solution (PBS containing 0.1% Triton X-100 and 3% BSA) for 1 h. Three PBS washes were performed (10 min each with shaking), before overnight incubation with primary antibodies (diluted in 3% BSA/PBS) at 4 °C. Following three additional PBS washes, cells underwent secondary antibody incubation for 1 h at room temperature. DAPI staining was performed for 2 min, followed by two PBS washes. Finally, we mounted the coverslips onto slides for examination using a Zeiss 710 NLO confocal microscope. The primary antibodies utilized included anti-POU5 F1 (…) and anti-NANOG (Bethyl, A300-397 A).

### mRNA Isolation and m^6^A quantification by ELISA

mRNA was isolated using mRNA Capture Beads (Vazyme, N401-01) following the manufacturer’s recommended protocol. mRNA m^6^A methylation levels were subsequently quantified using the MethylFlash™ m^6^A RNA Methylation ELISA Kit (EpigenTek, P-9010). Briefly, 200 ng of purified mRNA was immobilized onto ELISA wells, followed by sequential incubations with a capture antibody (60 min), detection antibody (30 min), and enhancer solution (30 min) at room temperature. After thorough washing, a colorimetric reaction was developed and the absorbance measured at 450 nm using a microplate reader. A standard curve was generated using the provided m^6^A positive control (0.02~0.4 ng/well) to enable precise quantification. The m^6^A methylation level was calculated as a percentage of total mRNA input using the formula: m^6^A% = (m^6^A amount/input mRNA amount) × 100%.

### mRNA stability measurements

To assess mRNA stability, D3 PNT cells carrying Ythdf2 knockout, along with control or STM2457-treated cells, were exposed to 50 nM actinomycin. At designated timepoints (0h, 4h, and 8 h), cells were harvested for analysis. Prior to constructing an RNA-seq library or performing reverse transcription for qPCR, add 0.1 uL of ERCC spike-in RNA control to each 1 ug of the RNA sample. Through RT-qPCR using gene-specific primers, we quantified the mRNA levels.

### Generation of chimeric mice

The generation of chimeric mice involved the injection of mouse rESCs into ICR blastocysts, which were subsequently transferred into pseudopregnant ICR females. To establish germline transmission, we conducted matings between F2 mice and ICR mice.

### NC/STM2457 PNT RNA-Seq analysis methods

#### Data preprocessing

The processed reads were aligned to the mouse Gencode (Mm10) transcriptome utilizing STAR.

Raw count data was read from a tab-delimited text file (PNTtotal_reads.txt) into R using the read.table() function. The dataset was then processed by creating a DESeqDataSet using the DESeq2 package with the experimental design based on the conditions (D3, D5, D7, EpiSC, OG2_ES, etc.).

#### Filtering and normalization

Data was pre-filtered by excluding genes with low expression across all samples (i.e., genes with row sums greater than 1 were retained). The data was normalized using the rlog transformation from DESeq2 to stabilize variance across conditions, and library size distributions were visualized before and after normalization.

#### PCA analysis

For the Principal Component Analysis (PCA), we used the rlog function from the DESeq2 package to transform the data, followed by the plotPCA function to generate PCA data.

#### Differential expression analysis

Differential gene expression analysis was performed on RNA-seq data. Genes were classified as upregulated (log_2_ fold-change ≥ 1), downregulated (log_2_ fold-change ≤ −1), or non-significant based on fold-change thresholds.

Gene ontology analysis was executed using clusterProfiler (version 3.18.0) and metascape(https://metascape.org/gp/index.html#/main/step1).

#### sgLuciferase/Ythdf2 PNT RNA-Seq analysis methods

Differential gene expression analysis was performed on RNA-seq data. Genes were classified as upregulated (log_2_ fold-change ≥ 1), downregulated (log_2_ fold-change ≤ −1), or non-significant based on fold-change thresholds.

An MA plot was generated using the ggplot2 package in R to visualize the results.

#### mRNA stability measurements RNA-Seq analysis methods

The processed reads were aligned to the mouse Gencode (Mm10) transcriptome and ERCC RNA spike-in sequence utilizing STAR.

We applied additional normalization using spike-in controls (ERCC) with the RUVg() function. Post-normalization, RLE and PCA plots were generated to assess the reduction of unwanted variation.

### MeRIP-seq and data analysis

RNA immunoprecipitation was performed as previously described (Shen et al. 2025). Briefly, non-ribosomal RNA samples were fragmented into about 150 nt fragments using Fragmentation Reagent (Thermo Fisher, AM8047), followed by end-repair with T4 Polynucleotide Kinase (Thermo Fisher, EK0031). The purified RNA fragments were ligated to adenylated 3’ adapters (NEB, E2610) using T4 RNA ligase 2 (NEB, M0373), with excess adapters removed by Lambda Exonuclease (NEB, M0262). Protein G beads (Thermo Fisher, 10004D) were conjugated with m6 A antibody (Cell Signaling Technology, 56593) through 1 h rotation at room temperature. After incubating the RNA fragments with antibody-coupled beads at 4 °C for 2 h, bound RNA was eluted using buffer RLT (QIAGEN, 79216). Subsequent library preparation included 5’ adapter ligation with T4 RNA ligase 1 (NEB, M0204), first-strand cDNA synthesis (HiScript III Kit, Vazyme R312), and PCR amplification (KAPA HiFi Hot Start Mix, KAPA Biosystems KK2602).

For data analysis, raw sequencing reads were quality-controlled using fastp (v0.24.0) (Chen 2023) with adapter trimming and quality filtering. Processed reads were aligned to the mm10 genome using HISAT2 (v2.2.1) (Kim et al. 2019) with parameters retaining multi-mapped reads for transposable element analysis while reporting single best alignments for ambiguous reads. Mitochondrial DNA-mapped reads were excluded during SAMtools (Li et al. 2009) processing, which removed duplicates and low-quality alignments (MAPQ ≥ 10). Normalized coverage tracks (RPKM) were generated using deepTools (Li et al. 2009) and visualized in IGV (Thorvaldsdottir et al. 2013). Peak calling employed a sliding window approach (100-nt window/50-nt step) (Liu et al. 2021), requiring ≥ fourfold IP/input enrichment, with adjacent enriched windows merged into final peaks.

## Supplementary Information

Supplementary Material 1. Figs. S1~S5.

Supplementary Material 2. qPCR Primer.

## Abbreviations

PNT: Primed to Naïve Transition
m⁶A: N6-methyladenosine
ESCs: Embryonic Stem Cells
EpiSCs: Epiblast Stem Cells
hESCs: Human Embryonic Stem Cells
mESCs: Mouse Embryonic Stem Cells
SCNT: Somatic cell nuclear transfer
BiPNT: BMP4-induced PNT
RNA-seq: RNA Sequencing
PE: Proximal enhancer
DE: Distal enhancer
rESCs: Reset embryonic stem cells
GO: Gene Ontology
NPT: Naïve to Primed transition
