# Supplementary material for: METTL3 inhibits primed-to-naïve transition of pluripotent stem cells through m6A-YTHDF2-pluripotency/Gstp1 mRNA degradation axis
Source: Cell Regen. 2025 May 27;14:19. doi: 10.1186/s13619-025-00241-1 (PMC12116966; doi:10.1186/s13619-025-00241-1)
Supplement: Supplementary file 1 — Supplementary Material 1. Figs. S1~S5. [file 13619_2025_241_MOESM1_ESM.docx]

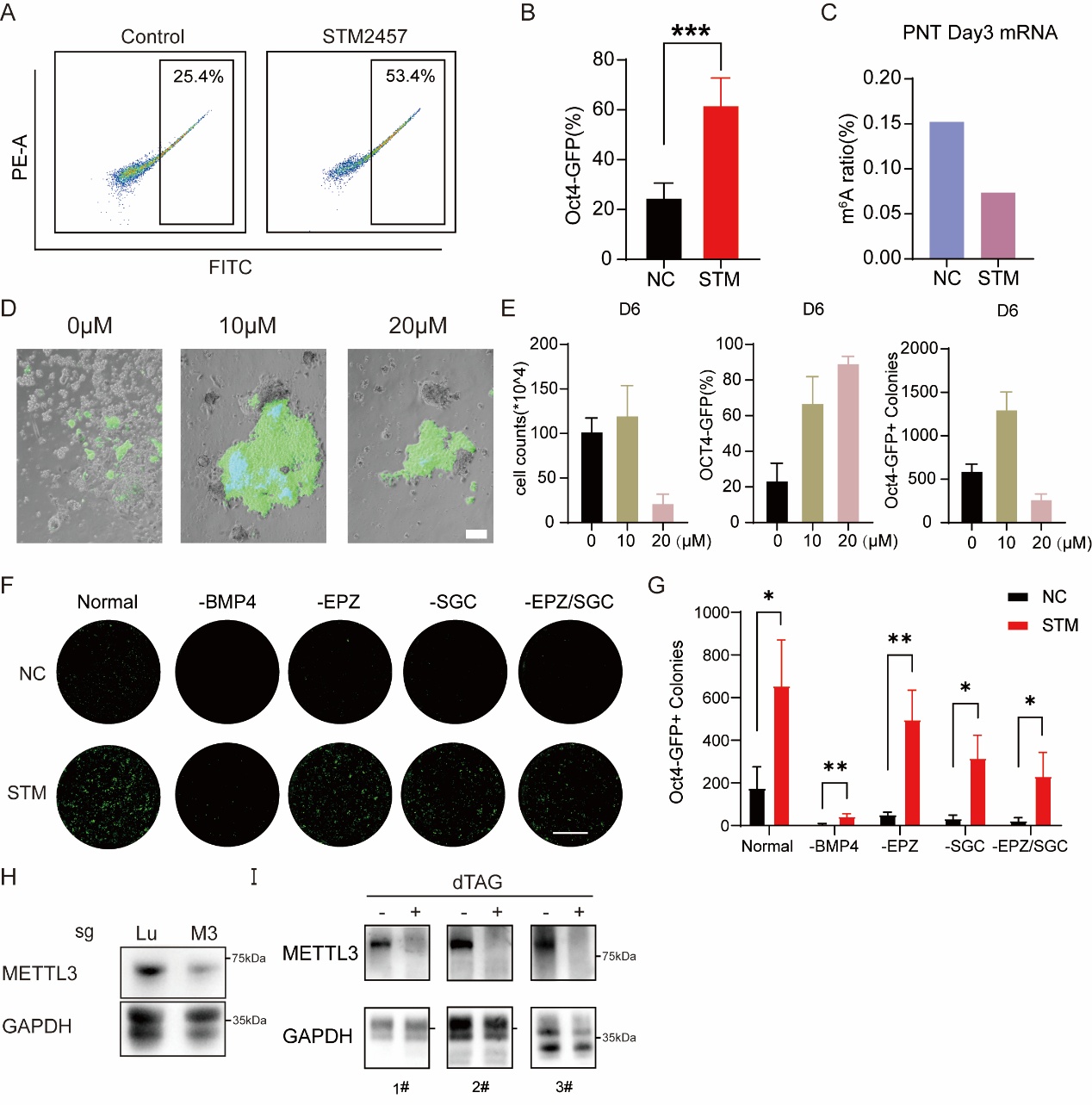


**Figure S1. Related to Figure 1.**

(A and B), Flow cytometry analysis of GFP+ cells at day 8 of PNT with or without STM2457 treatment. A, Representative FACS plots. B, Quantification of GFP+ cells. (n = 3, error bar, s.d; unpaired t-test, *p < 0.05, **p<0.01, ***p<0.001)

(C) m^6^A levels in mRNA measured by ELISA after 3 days of STM2457 treatment compared to control.

(D) Representative fluorescence images showing PNT efficiency at day 6 following treatment with varying concentrations of STM2457. Scale bars, 100 μm.

(E) Quantification of total cell number, Oct4-GFP+ cell percentage, and Oct4-GFP+ colony count at day 6 of PNT across different STM2457 concentrations. (n = 2, error bar, s.d)

(F) Oct4-GFP fluorescent scanning image of whole 24-well plates showing PNT efficiency at day 6 with and without STM2457 treatment following removal of different small molecules. Scale bars, 5 mm.

(G) Quantification of Oct4-GFP+ colonies under conditions described in (F). (n = 3, error bar, s.d; unpaired t-test, *p < 0.05, **p<0.01, ***p<0.001)

(H) Western blot analysis of METTL3 protein expression in EpiSCs following CRISPR-Cas9-mediated targeting. GAPDH serves as loading control.

(I) Western blot analysis of METTL3 protein levels in METTL3-FKBP EpiSCs treated with or without dTAG. GAPDH serves as loading control. (n = 3)


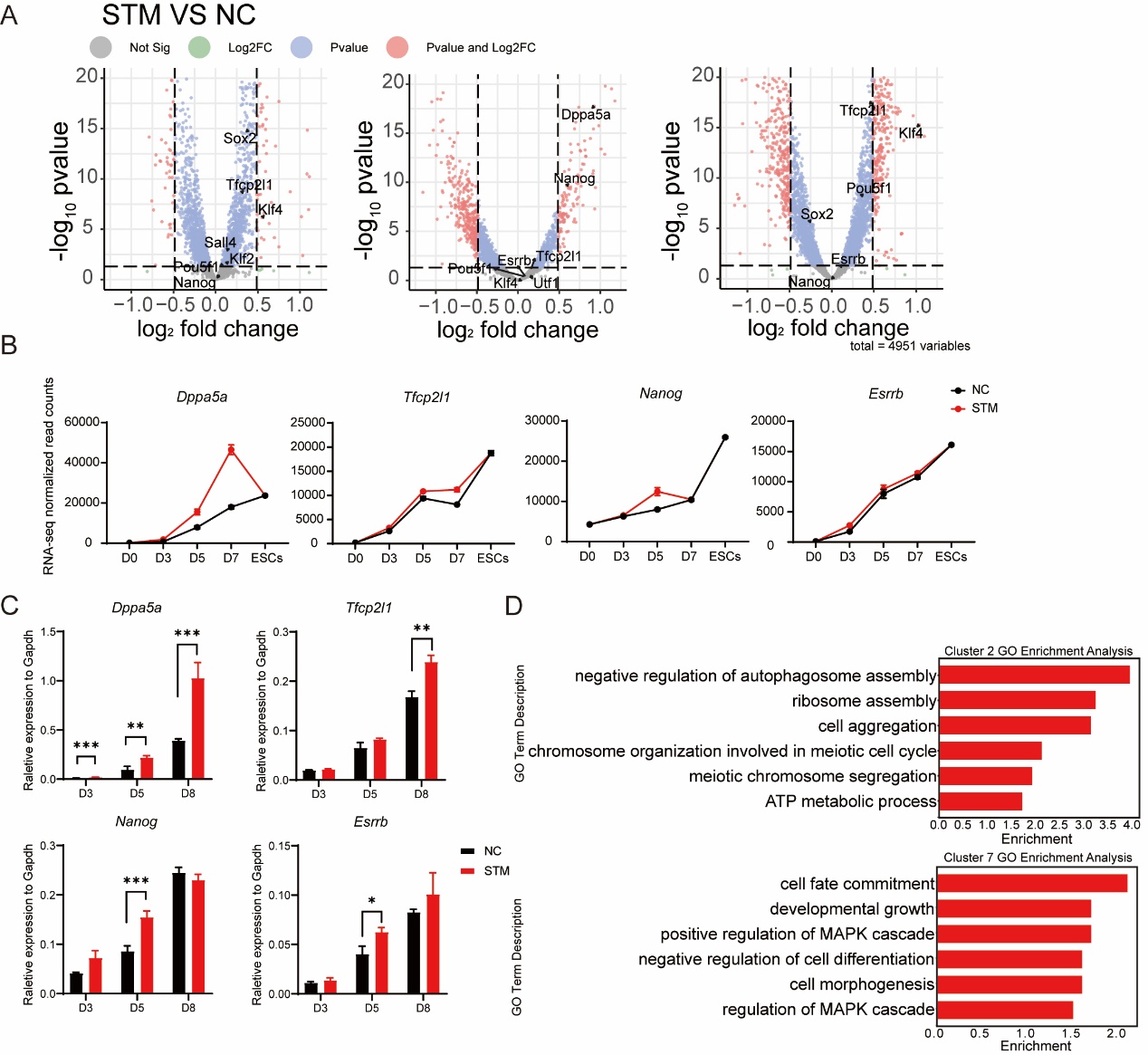


**Figure S2. Related to Figure 2.**

(A) Dot plot showing differentially expressed genes (DEGs) from RNA-seq results of D3/5/7 PNT, with or without STM2457 treatment. Naïve pluripotent genes are labeled.

(B) RNA-seq analysis of the expression levels of the selected pluripotent genes.

(C) RT-qPCR analysis of the expression of selected naive pluripotent genes in D3/5/7 PNT, with or without STM2457 treatment. Data normalized to GAPDH. (n = 3, error bar, s.d; unpaired t-test, *p < 0.05, **p<0.01, ***p<0.001)

(D) GO analysis of genes in cluster 2 and cluster 7 of Figure 2E.


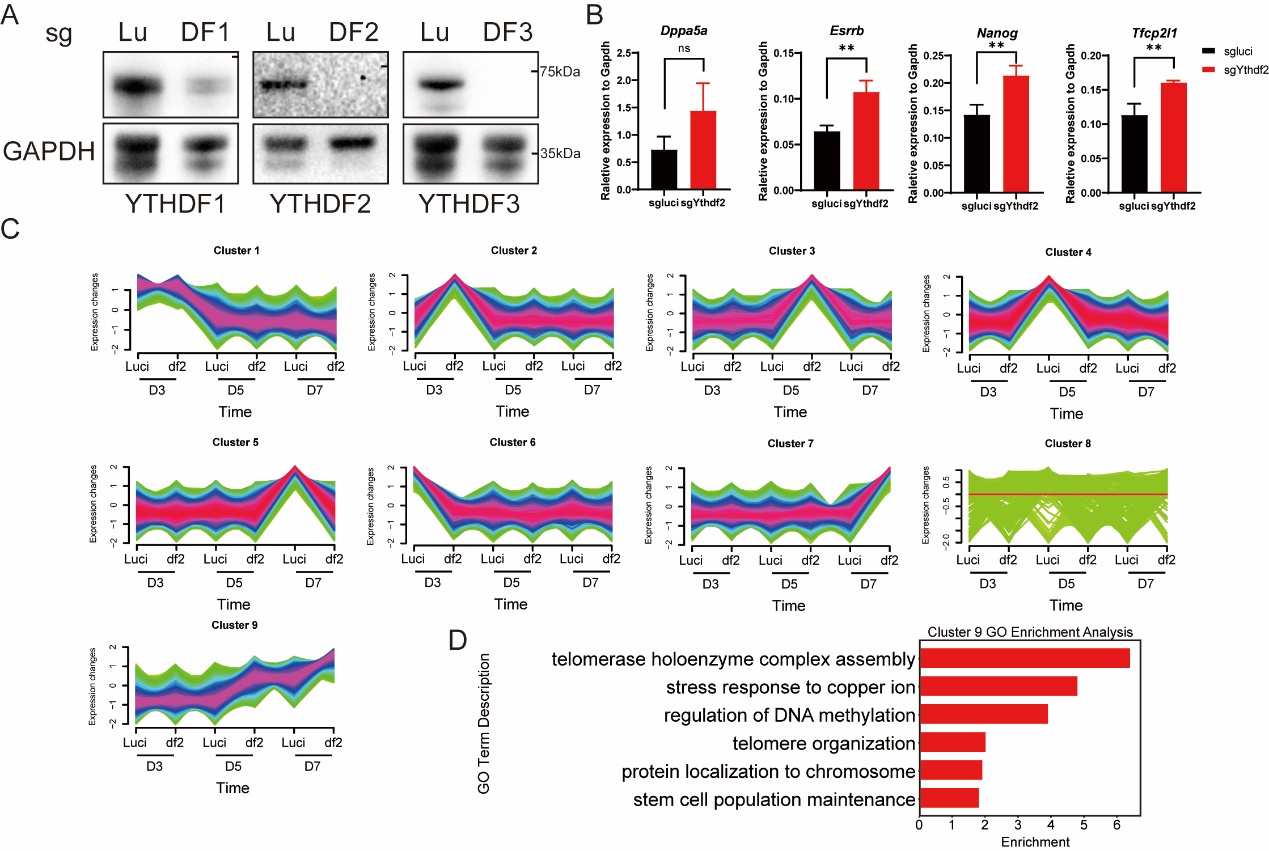


**Figure S3. Related to Figure 3.**

(A) Western blot analysis confirming successful knockout of YTHDF1, YTHDF2, and YTHDF3 proteins. GAPDH serves as loading control.

(B) RT-qPCR analysis of naïve pluripotency markers in control and YTHDF-deficient cells during PNT. Data normalized to GAPDH. (n = 3, error bar, s.d; unpaired t-test, *p < 0.05, **p<0.01, ***p<0.001)

(C) Mfuzz clustering analysis showing nine distinct gene expression patterns during PNT in control versus *Ythdf2*-knockout cells.

(D) GO analysis of genes in cluster 9 showing enrichment of pluripotency maintenance pathways.


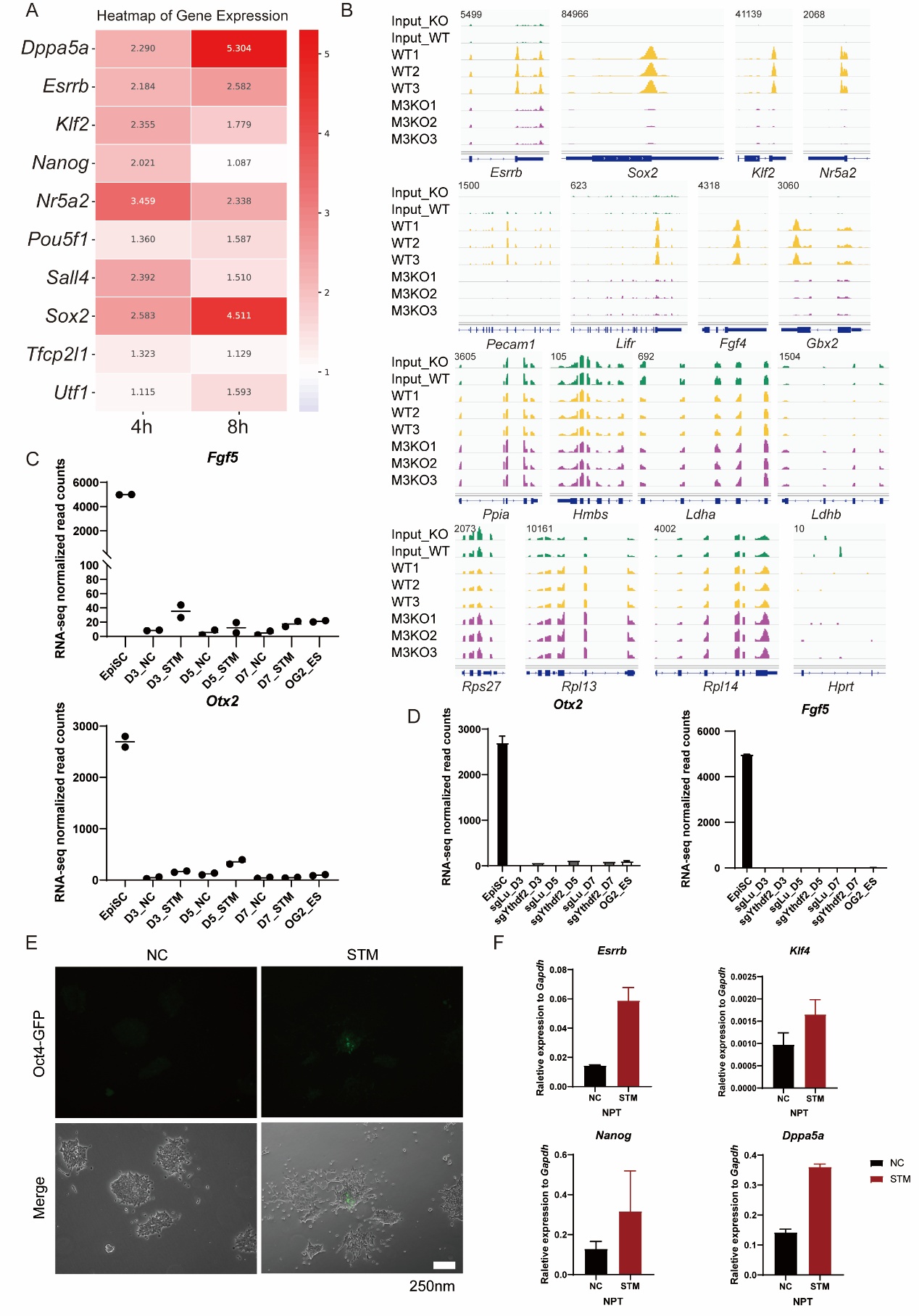


**Figure S4. Related to Figure 4.**

(A) Heatmap depicting the ratio of mRNA lifetime between STM-treated and Control (NC). Data were derived from RNA-seq. Cells were treated with Actinomycin D (ActD) and samples were collected at 0h, 4h, and 8h.

(B) m^6^A peak coverage on specific naïve pluripotent transcripts and housekeeping transcripts in published data.

(C) RNA-seq analysis of the expression levels of the indicated primed genes. Data were from PNT RNA-seq with or without STM treatment.

(D) RNA-seq analysis of the expression levels of the indicated primed genes. Data were from PNT RNA-seq where *Ythdf2* and *Luciferase* were knocked out using CRISPR-Cas9. The ES and EpiSC data were obtained from PNT RNA-seq with or without STM treatment, as the same in (C).

(E) Fluorescence Images of EpiSC Induced from OG2-ES. (bar=250nm)

(F) RT–qPCR analysis of the expression of specific naive pluripotent mRNAs in EpiSC Induced from OG2-ES. (n=3, error bar, s.d)


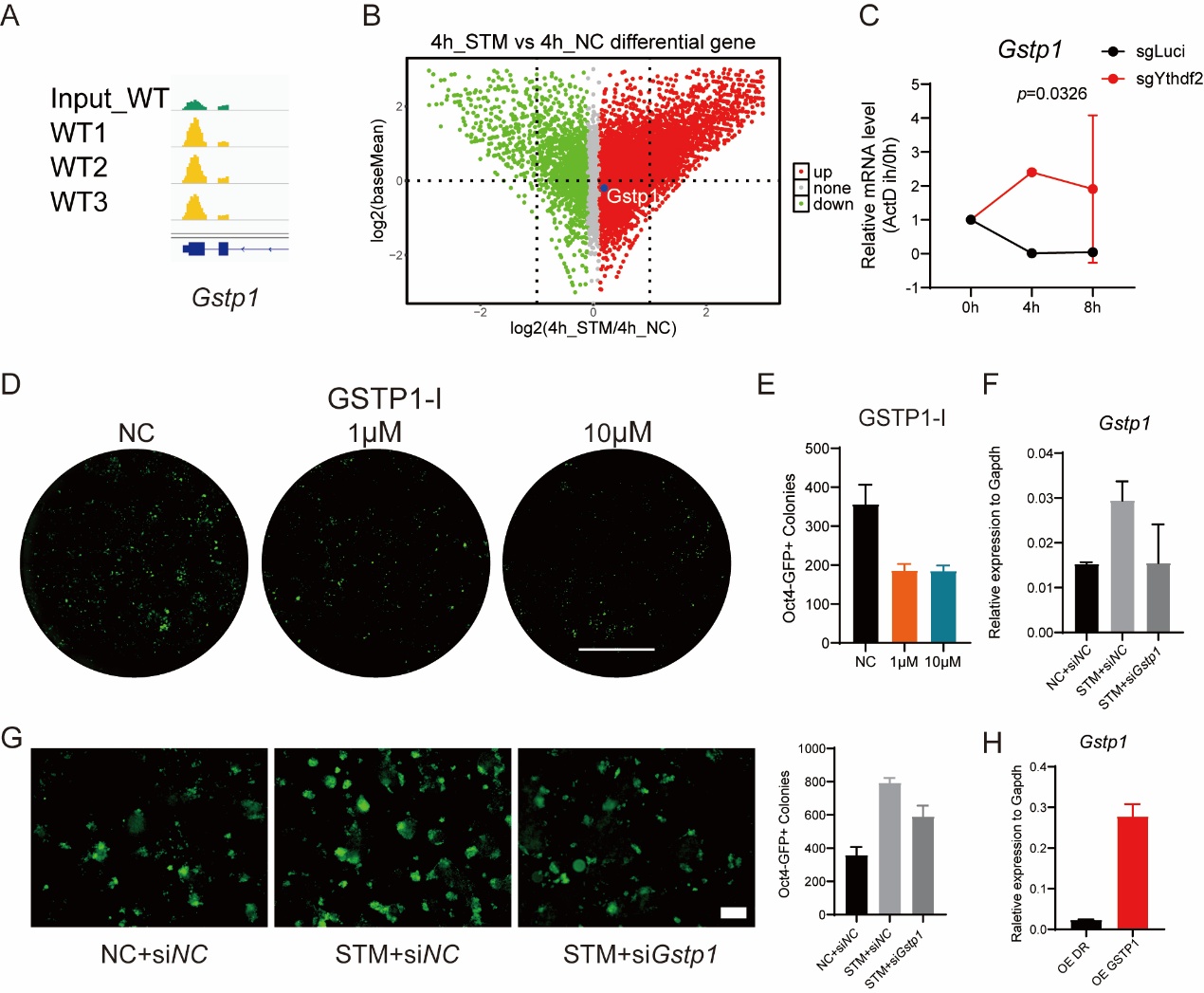


**Figure S5. Related to Figure 5.**

(A) m^6^A peak coverage on *Gstp1* mRNAs in published data.

(B) mRNA lifetime analysis of RNA-seq data in D3 PNT with or without STM Treatment, Cells were treated with Actinomycin D (ActD) and samples were collected at 0h, 4h. Red dots represent genes with inhibited degradation. *Gstp1* gene are labeled.

(C) mRNA lifetime analysis of *Gstp1* from RT-qPCR in D3 PNT with *Ythdf2* or *luciferase* (as a Control) knocked out, Cells were treated with Actinomycin D (ActD) and samples were collected at 0h, 4h, and 8h. (n = 3, error bar, s.d; Two-way ANOVA with p‑values calculated for the column factor)

(D) Oct4 - GFP fluorescent scanning image of the whole 24 - well plates on the 7th day of PNT treated with different concentrations of GSTP1 - I. Scale bars, 5 mm

(E) Number of GFP+ colonies of D7 PNT related to (C). (n = 2, error bar, s.d)

(F) RT–qPCR analysis of the expression of *Gstp1* gene treated with STM2457 and knockdown by siRNA. (n = 2, error bar, s.d)

(G) Left: Oct4-GFP fluorescent image showing PNT efficiency with STM2457 treatment and knockdown by siRNA. Scale bars, 250 nm. Right: Quantification of GFP+ colonies. Data are mean ± s.d., n = 3 independent experiments.

(H) RT–qPCR analysis of the expression of *Gstp1* gene in EpiSC with overexpressed *Gspt1* or *Ds-red*. (n = 2, error bar, s.d)
